# Terbium sisters: current development status and upscaling opportunities

**DOI:** 10.3389/fnume.2024.1472500

**Published:** 2024-10-11

**Authors:** Anzhelika N. Moiseeva, Chiara Favaretto, Zeynep Talip, Pascal V. Grundler, Nicholas P. van der Meulen

**Affiliations:** ^1^ Center for Radiopharmaceutical Sciences, PSI Center for Life Sciences, Villigen PSI, Switzerland; ^2^ Radiopharmacy and Cyclotron Department, IRCCS Sacro Cuore Don Calabria, Negrar di Valpolicella, Italy; ^3^ PSI Center for Nuclear Engineering and Sciences, Villigen PSI, Switzerland

**Keywords:** terbium, theragnostics, radiolanthanides, production capabilities, nuclear reactions

## Abstract

The interest in terbium radionuclides, which can be used in nuclear medicine, has increased tremendously over the last decade. Several research studies have shown the potential of four terbium radionuclides ^149,152,155,161^Tb both for cancer diagnosis as well as therapy. The comparison of ^161^Tb and ^177^Lu showed ^161^Tb as the preferred candidate not only for standard radiotherapy, but also for the treatment of minimal residual disease. Nevertheless, among the terbium sisters, currently, only ^161^Tb has an established production protocol where its no-carrier-added form is obtained via neutron irradiation of enriched ^160^Gd targets. The other terbium radioisotopes face challenges related to production capacity and production yield, which currently restricts their use in nuclear medicine. The purpose of this review is to report on recent research on the production and separation of terbium sisters and to assess the prospects for upscaling their production for nuclear medicine applications.

## 1 Introduction

According to World Health Organization, about one in five people develop cancer in their lifetime, while approximately one in nine men and one in 12 women die from the disease (1). The share of nuclear medicine in cancer treatment has seen significant growth in the last few decades. This progress is largely due to advancements in radiopharmaceutical delivery to cancer cells, enhancing both diagnosis and therapy. Contemporary radiopharmaceuticals with radiometals usually consist of the vector–chelator–radionuclide framework to offer flexibility in the choice of radionuclides. Currently, there are only a few radiometal-based radiopharmaceuticals that have been approved by medical authorities, namely, Lutathera [(^177^Lu)Lu-DOTA-TATE], Pluvicto [(^177^Lu)Lu-PSMA-617], Octreoscan [(^111^In)In-DTPA-pentetreotide], [^67^Ga]Ga-citrate, Netspot [(^68^Ga)Ga-DOTA-TATE], [^68^Ga]Ga-DOTA-TOC, Locametz/Illuccix [(^68^Ga)Ga-PSMA-11], Detectnet [(^64^Cu)Cu-DOTA-TATE], and Zevalin ([^90^Y]Y-ibritumomab-tiuxetan (2). However, there can be a much broader choice of radiometals, suitable for cancer treatment or diagnosis, according to their nuclear properties. Furthermore, radiometals’ application in nuclear medicine fosters innovative strategies (such as imaging, treatment with α- or Auger-electron emitters, and theragnostics) that could enhance the role of radiopharmaceuticals in cancer treatment and diagnosis.

**Figure 1:**
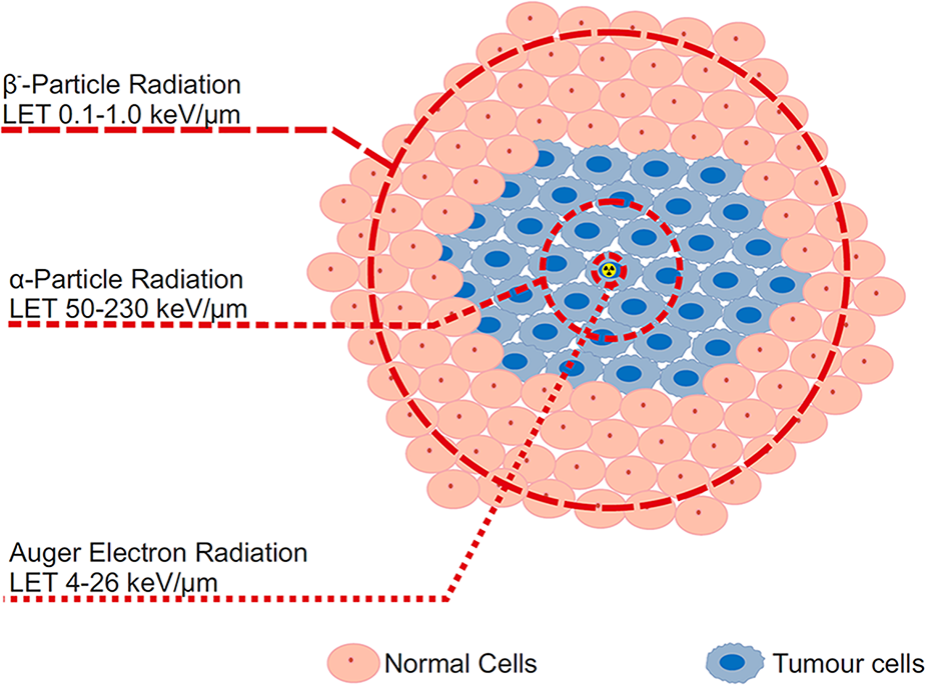
Illustration demonstrating linear energy transfer (LET) range of α-particle, β-particle, and Auger election radiation within tumour cells from a specific radioactive source (indicated by the radioactive symbol).

The current trend in Radioligand Therapy (RLT) revolves around the use of ^177^Lu (T_1/2_ = 6.65 d), as a therapeutic β^−^-emitter, e.g., in radiopharmaceuticals such as Pluvicto and Lutathera (2). The use of this radiolanthanide in the clinical setting has encouraged research into other radiolanthanides/actinides that may have therapeutic capabilities. While ^177^Lu is currently seen as the most prominent radiometal in RLT, it does have its shortcomings, as its curative effect has not been as significant as expected (3). Particularly, the wide range of β^−^-particles in biological tissue—up to 12 mm—implies a low linear energy transfer (LET, ∼0.2 keV/µm), which might not effectively damage targeted cells (Figure 1). However, based on theoretical data, it was calculated that α-emitters are about two orders of magnitude more effective in killing cells (4). ^149^Tb, a potential therapeutic radionuclide due to an emission of α-particles, has a LET of 142 keV/µm, while the penetration range itself is only 28 µm (5). This indicates that the treatment can be more effective and that healthy cells around the tumour should suffer less. Thus, more attention is being focused on radionuclides with lower ranges of emitted particles and greater LET. Conversion/Auger electron (LET ∼20 keV/µm for ^161^Tb) and α-emitters are coming to the fore in production and nuclear medicine application possibilities (6).

Delivering such short-range radiation to the cell nucleus seems to be essential, presenting several challenges in radiopharmaceutical delivery. However, it appears that the membrane is a more sensitive target than the cytoplasm for the dense ionization produced by Auger electrons (7). This indicates that, even in the absence of nuclear localization, the proximity of an Auger-electron emitter to the cancer cell membrane may still significantly enhance the therapeutic efficacy.

Another important aspect of radiometals is the synergistic properties of the radioisotopes, which can be effectively utilized in the theragnostic concept. It is particularly promising where diagnostic imaging and therapeutic interventions apply the same radioligand, emitting particles suitable both for therapy and diagnostic [= theragnostic (8)] purposes (9). For example, ^149^Tb can be applied in targeted α-therapy (TAT) and could potentially provide visualization by positron emission tomography/computed tomography (PET/CT), thus, enabling efficient dosimetry (10). In alternative scenarios, sets of radioisotopes are employed wherein one radionuclide fulfills a diagnostic function, while the other serves a therapeutic purpose. According to this principle, a triplet of radioisotopes, namely, ^123^I (EC), ^124^I (β^+^), and ^131^I (β^−^) is already actively used (11), and other sets, such as ^43^Sc (β^+^), ^44^Sc (β^+^), ^47^Sc (β^−^) and ^61^Cu(β^+^), ^64^Cu (β^+^ 61.5%, β^−^ 38.5%), ^67^Cu (β^−^), are being actively studied. Another example would be diagnostic ^152^Tb for PET or ^155^Tb (T_1/2_ = 5.32 d, *ε*) for single photon emission computed tomography (SPECT) combined with ^161^Tb (T_1/2_ = 6.95 d, β^−^ 100%, conversion and Auger-electrons) for therapy.

**Figure 2:**
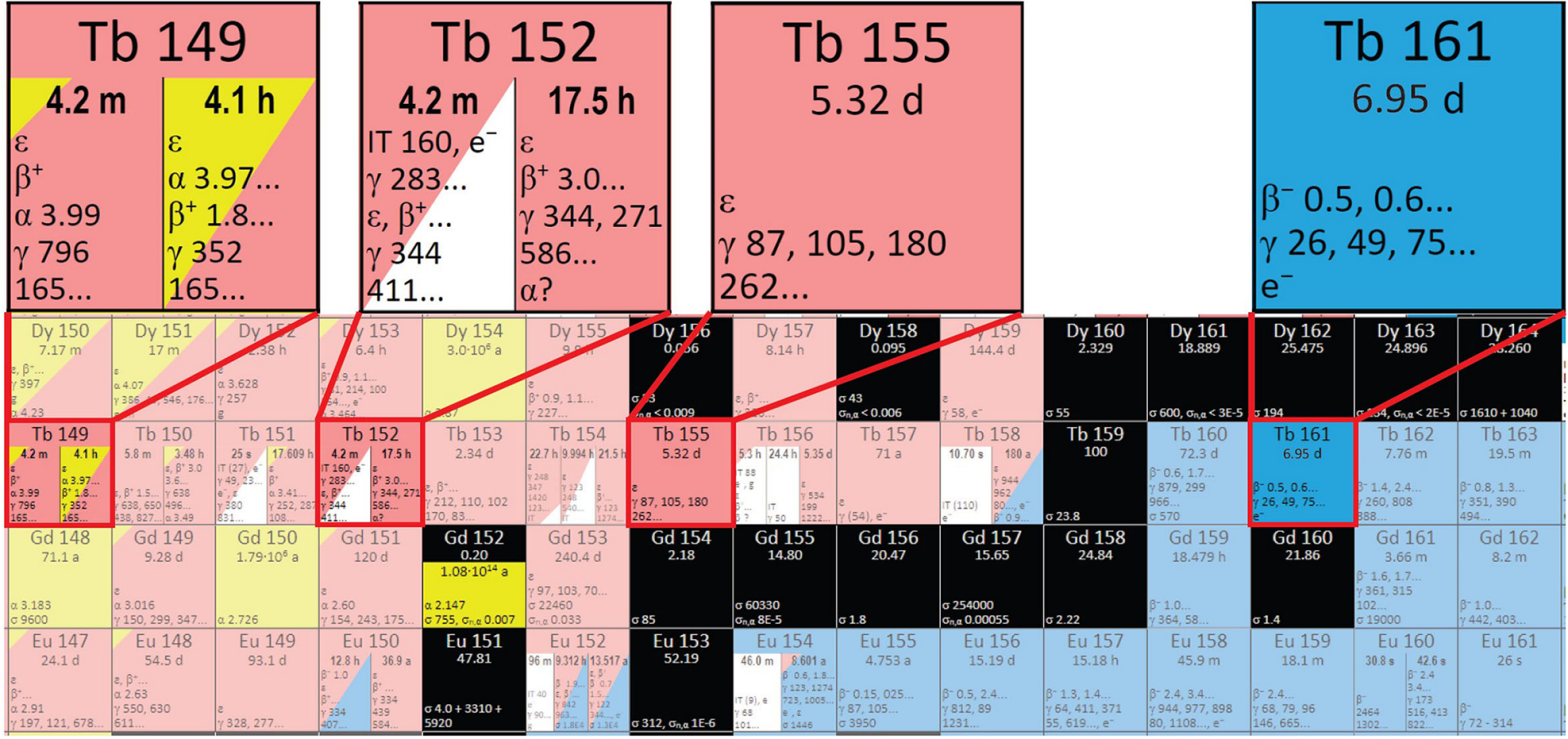
A section of the Karlsruhe nuclide chart showing the region containing the terbium sisters (adapted from www.nucleonica.com).

Terbium radioisotopes meet all the requirements of modern nuclear medicine, mentioned above. ^149^Tb exhibits theragnostic properties according to its nuclear data (with the possibility to use it for PET, as well as TAT). ^152^Tb has performed well in PET/CT imaging and has been applied in preclinical testing, as well as first-in-human applications (12–14). ^155^Tb has soft γ-lines similar to ^99m^Tc, which allows it to be used in SPECT (15). ^161^Tb is the closest analogue of the very popular therapeutic β^−^-emitter ^177^Lu in terms of its nuclear and chemical properties; however, it is more effective due to the additional effect of conversion and Auger-electron emission, should it be applied with the correct targeting agent (16, 17). ^161^Tb and ^155^Tb can be obtained with simply achievable (n,γ) (18) and (p,n)/(p,2n)/(d,2n) nuclear reactions (Figure 2) (19, 20), respectively. ^149^Tb and ^152^Tb, however, are located further away from the stable nuclides in the nuclide chart; therefore, it is more challenging to produce them. Alternative methods may be used for their production, such as α-induced irradiations of europium targets (21, 22).

Despite the proven effectiveness of the terbium sisters (23) in animal experiments, their production is associated with serious limitations, thereby, preventing clinical studies. To date, only ^161^Tb can be produced in quantities and purity for clinical application, where its no-carrier-added (n.c.a.) form is obtained via neutron irradiation of enriched ^160^Gd targets (24). The process has recently been commercialized, indicating its potential for future application (25). The other terbium radioisotopes have significant challenges related to production capacity and production yield, requiring either ultra-enriched target materials (for example, >99% enriched ^152^Gd) or middle- and high-energy particle accelerators paired with Isotope Separation On-Line (ISOL) facilities, whose availability is limited. Only a few institutions worldwide have worked on new methods for terbium sisters’ production (Tables 1–4). Of those, 22 have published articles about terbium production in the last decade. The main results will be further discussed in this review.

**Table 1:** Main routes and facilities for ^161^Tb production.

Reaction	TTY	Radionuclidic purity	Target material availability	Production Facility	Chemical separation performed?	Recent publication	Reference
^nat^Gd(d,x)	Low	Low	Available	VUB (CGR 560)	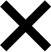	2014	(26)
				LLN (Cyclone 90)			
				ATOMKI (MGC-20)	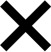	2016	(27)
				NIRS (AVF-930)			
^160^Gd(d,n)	Medium	86%	Expensive	VUB (CGR 560)	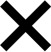	2013	(28)
				LLN (Cyclone 90)			
^nat^Dy(γ,x)	Low	Low	Available	SINP MSU (RTM-55)	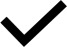	2023	(29)
^160^Gd(n,γ)	High	99.9%	Expensive	PSI (SINQ)	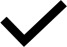	2019	(18)
				HZB (BER II)	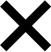	2011	(30)
				TUM (FRM II)	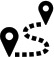 to PSI		
				ILL (RHF)	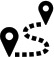 to PSI	2023	(18, 30–32)
				SCK CEN (BR2)	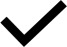 / 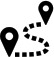 to TRIUMF	2023	(33–35)
				CV Řež (LVR 15)	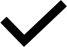	2022	(36)
				CRDNT (Triga 2000)	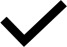	2020	(37, 38)
				NECSA (SAFARI-1)	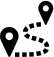 to PSI	2023	(24, 32)

**Table 2:** Main routes and facilities for ^155^Tb production.

Reaction	TTY	Radionuclidic purity	Target material availability	Production Facility	Chemical separation performed?	Recent publication	Reference
^nat^Gd(p,x)	High	Low	Available	AEC (Inselspital medical cyclotron)	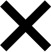	2022	(39)
				ARRONAX	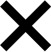	2020	(40)
				NIRS (AVF-930)	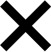	2012	(41)
				iThemba LABS (SSC)			
^155^Gd(p,n)	Medium	94%	Expensive	PSI (IP2)	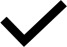	2021	(19)
				UniBe LHEP (Inselspital medical cyclotron)	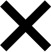	2022	(39)
^156^Gd(p,2n)	High	92%	Expensive	PSI (IP2)	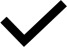	2021	(19)
^nat^Tb(p,x)	High	99.9%^a^	Available	PSI (IP2)	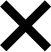	2022	(42, 43)
				LANL (LANSCE)	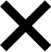	2012	(44)
				iThemba LABS (SSC)	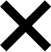	2014	(45)
^nat^Dy(p,x)	Low	Low	Available	LLN (Cyclone 90)	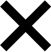	2015	(46)
				VUB (CGR 560)	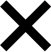	2013	(47)
				NPI CAS (U-120M)	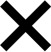	2022	(48)
				KIRAMS (MC 50)	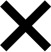	2020	(49)
^nat^Gd(d,x)	High	Low	Available	ARRONAX	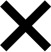	2022	(50, 51)
				VUB (CGR 560)	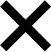	2014	(26)
				LLN (Cyclone 90)			
				ATOMKI (MGC-20)	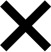	2016	(27)
				NIRS (AVF-930)			
				RIKEN (AVF cyclotron)	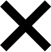	2023	(52)
^155^Gd(d,2n)	High	89%	Expensive	ARRONAX	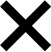	2023	(20)
^153^Eu(α,2n)	Medium	66%	Available	SRC-FMBC (EA-25)	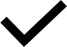	1977	(53)
^nat^Gd(α,x)	Medium	94^a^	Available	JRC (IK III/Zyklotron)	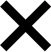	1996	(54)
				Kurchatov Institute (U-150)	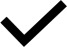	2022	(55)
				RIKEN (AVF cyclotron)	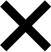	2022	(56)
^155^Gd(α,x)	High	99.9%^a^	Expensive	Kurchatov Institute (U-150)	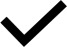	2023	(57)
^nat^Dy(γ,x)	Low	Low	Available	SINP MSU (RTM-55)	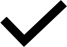	2023	(29)
^156^Dy(γ,x)	Low	99.98%	Ultra-expensive	SRC-FMBC (EA-25)	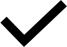	1981	(58)
^nat^Ta(p,x)	Low	99.9%^b^	Available	CERN (ISOLDE)	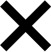 / 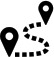 to PSI	2020/2014	(15, 59)
				CERN (MEDICIS)	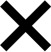 / 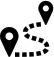 to NPL	2019	(60)
				LNS (proton accelerator)	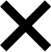	2002	(61)
				TRIUMF	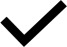	2023	(34)

^a^ High radionuclidic purity is achievable after rapid separation of the Dy fraction from an irradiated target.

^b^ High radionuclidic purity is achievable after ISOL separation methods.

**Table 3:** Main routes and facilities for ^152^Tb production.

Reaction	TTY	Radionuclidic purity	Target material availability	Production Facility	Chemical separation performed?	Recent publication	Reference
^nat^Gd(p,x)	Medium	Low	Available	ARRONAX	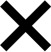	2020	(40)
				NRC AEA (ICF MGC-20)	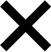	2007	(62)
				NIRS (AVF-930)	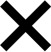	2012	(41)
				iThemba LABS (SSC)			
^152^Gd(p,n)	High	99%	Only low enrichment available	TUM, LMU (tandem accelerator of MLL Garching)	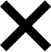	2020	(63)
				ATOMKI (MGC-20)	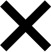	2015	(64)
^155^Gd(p,4n)	High	Low	Expensive	iThemba LABS (SSC)	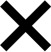	2014	(45)
^nat^Gd(d,x)	Low	Low	Available	ARRONAX	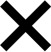	2016	(50)
				LLN (Cyclone 90)	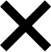	2014	(26)
^nat^Dy(d,x)	Low	Low	Available	LLN (Cyclone 90)	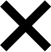	2015	(46)
^151^Eu(α,3n)	High	80%	Available	Kurchatov Institute (U-150)	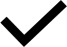	2023	(22)
^nat^Ta(p,x)	Low	99.9%^a^	Available	CERN (ISOLDE)	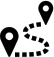 to PSI	2019	(12, 14, 65)
^nat^Nd(^12^C,x)	Medium	Medium	Available	NPD-BARC (BARC-TIFR Pelletron Accelerator)	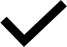	1999	(66)
				ANU (14 UD)	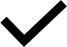	2001	(67–69)

^a^ High radionuclidic purity is achievable after ISOL separation methods.

**Table 4:** Main routes and facilities for ^149^Tb production.

Reaction	TTY	Radionuclidic purity	Target material availability	Production Facility	Chemical separation performed?	Recent publication	Reference
^nat^Gd(p,x)	Low	Low	Available	ARRONAX	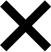	2020	(40)
^152^Gd(p,4n)	Medium	Low	Only low enrichment available	iThemba LABS (SSC)	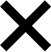	2014	(45)
^151^Eu(^3^He,5n)	Medium	43%	Available	Kurchatov Institute (U-150)	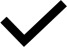	2020	(21)
^nat^Ta(p,x)	Low	99.9%^a^	Available	CERN (ISOLDE)	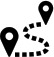 to PSI	2024	(70)
				FZ Jülich (COSY synchrotron)	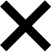	2020	(71)
				LBNL (bevatron, 184-in. cyclotron)	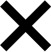	1964	(72)
				UChicago (Fermi Institute cyclotron, Synchrocyclotron)			
^nat^Nd(^12^C,x)	Medium (can be increased with ^142^Nd)	Medium	Available	LNR JINR (U-200)	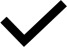	2003	(73)
				NPD-BARC (BARC-TIFR Pelletron Accelerator)	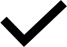	1999	(66)
				ANU (14 UD)	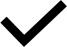	1997	(67, 74)
				LBNL (heavy-ion linear accelerator)	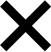	1963	(75)

^a^ High radionuclidic purity is achievable after ISOL separation methods.

## 2 Production of terbium radioisotopes at large research facilities around the world

### 2.1 Neutron irradiation

The most effective route to produce neutron-rich nuclides is the (n,γ) nuclear reaction thanks to their relatively high cross-sections and potential of utilizing a high neutron flux. However, to produce n.c.a. radionuclides, the use of an indirect production route, should one exist, is advantageous. An example of this type of method was explored and implemented in ^161^Tb production. The indirect route ^160^Gd(n,γ)^161^Gd → ^161^Tb is attractive due to its simplicity and product quality (30). The enriched ^160^Gd target is irradiated with neutrons, which induce ^161^Gd production (T_1/2_ = 3.66 min, β^−^) with a cross section of 1.4 b. ^161^Gd quickly decays into ^161^Tb and, upon completion of the separation of Tb from the gadolinium target, a high-quality product with radionuclidic purity (RNP) > 99.99% is obtained. The main impurity is ^160^Tb (T_1/2_ = 72.3 d, β^−^), which is produced by the activation of ^159^Tb, present as impurity in the target material. Initially, a collective of authors from the Technical University of Munich (TUM), the Paul Scherrer Institute (PSI), Institut Laue-Langevin (ILL), and University of Bern presented a possibility to produce up to 15 GBq (up to 80% of the available activity) after 14-day irradiation at BER II/FRM II of up to 40 mg ^160^Gd_2_O_3_ (98.2% ^160^Gd) and subsequent separation (30). ^161^Tb–DOTA-peptide could be prepared with >99% reaction yield by incubating ^161^Tb and DOTA-TATE, corresponding to a ^161^Tb-to-DOTA-TATE molar ratio of 1:12 (30). To date, several facilities have been producing ^161^Tb via this route (Table 1). Tens of GBq ^161^Tb were produced in the collaboration between ILL, the South African Nuclear Energy Corporation (NECSA), and PSI (24). The product was obtained at an activity concentration of 11–21 MBq/*μ*l with ≥99% radionuclidic and radiochemical purity (24). Radiolabelling of PSMA-617 and other DOTA-based targeting compounds with ^161^Tb was achieved at molar activities up to 100 MBq/nmol at a radiochemical purity of ≥98%, demonstrating the high quality of the radionuclide (76). It has been stipulated that 7-day irradiation of 10 mg of ^160^Gd_2_O_3_ (98.2% ^160^Gd) targets at SCK.CEN's BR2 reactor typically produces 7–10 GBq of ^161^Tb (33). Radiolabelling of crown-αMSH with ^161^Tb was achieved at molar activities up to 144.9 MBq/nmol at a radiochemical purity ≥99% (33). Lately, ^161^Tb production has been presented by other research groups, such as the Center for Applied Nuclear Science and Technology—National Nuclear Energy Agency of Indonesia (BATAN), and Czech Technical University in Prague, FNSPE. The Indonesian group stated that a quartz ampoule with 5 mg of ^160^Gd_2_O_3_ target was irradiated with thermal neutron flux ∼10^13^ n·cm^−2^·s^−1^ during ∼3-day irradiation at the Bandung Triga 2,000 Reactor (37). Unfortunately, the ^161^Tb yield was not reported. The Czech group did not report it either after irradiations in the nuclear reactor LVR 15 (CV Řež) (36).

The ^161^Tb production process can be scaled up by increasing the target mass and aiming for higher neutron fluxes. However, the availability of high flux reactors is a critical point due to the shutdown of older reactors (for example, High Flux Beam Reactor and Brookhaven Medical Research Reactor at BNL), thus, requiring the construction of new ones to meet the growing demand for neutron-rich radionuclides. At least nine organizations produced ^161^Tb using the ^160^Gd(n,γ)^161^Gd → ^161^Tb production route (Table 1). There are likely more working on preclinical activities, however. It is expected to increase drastically over the next decade, due to the growing frequency of clinical studies involving ^161^Tb. According to ClinicalTrials.gov, there are three approved clinical trials at the time of writing (77–79).

### 2.2 Light-ion and photon-induced nuclear reactions

The most common proton and deuteron-induced reactions on gadolinium (natural and enriched) targets were explored (19, 26, 27, 39–41, 45–47, 50, 62–64). Generally, ^152,155^Tb production routes were studied, however, the ^152^Gd(p,4n)^149^Tb nuclear reaction was also investigated (45).

The most optimal reactions for ^152^Tb production are ^152^Gd(p,n) [42.8 kBq after 2 h irradiation with 12 MeV protons, at an average current of 0.9 μA (63)] and ^155^Gd(p,4n) [cross-section peak 900 mb at an energy of about 39 MeV (45)]. Despite promising results, they are complicated to realize. For example, the target material enriched in ^152^Gd is only commercially available as 30% enrichment due to its low natural abundance (0.20%). Nuclear reactions on the other gadolinium nuclides drastically decrease the RNP. When irradiating ^155^Gd with protons, there are two disadvantages. Firstly, the enrichment of available ^155^Gd target material does not exceed 93%. So, as with the ^152^Gd target, parallel reactions on the other gadolinium isotopes negatively affect the RNP. Secondly, the (p,4n) reaction is accompanied by (p,3n) reaction in the lower energies and (p,5n) reaction in the higher energies of protons. The contribution of these reactions can be varied, but in conjunction with the first disadvantage, it may require an electromagnetic isotope separation for nuclear medicine application of ^152^Tb (45).

Two proton-induced nuclear reactions for ^155^Tb production were studied at the Paul Scherrer Institute (Switzerland) and the Bern medical cyclotron (Inselspital University Hospital Bern), namely, ^155^Gd(p,n) and ^156^Gd(p,2n). In the case of ^155^Gd irradiation, the cross section presents a peak at around 11.5 MeV, corresponding to 447 mb, while in the case of ^156^Gd, the cross-section peak is about 1 b at 18 MeV (39). Production yields of ^155^Tb were calculated as well. The yield using the reaction ^155^Gd(p,n)^155^Tb, a target mass of ∼40 mg and beam entry energy ∼10.3 MeV equals 0.42 ± 0.26 MBq/µAh. Irradiations of ^156^Gd gave more promising results. The yield of the reaction ^156^Gd(p,2n)^155^Tb with target mass ∼40 mg and beam entry energy ∼22.8 MeV equals 3.28 ± 0.65 MBq/µAh (19). Unfortunately, ^156^Tb was produced as a radionuclidic impurity in both scenarios (6% for ^155^Gd irradiation and 8% for ^156^Gd irradiation). It is an inevitable impurity due to its comparable half-life (5.35 d) and target material enrichment. Supposedly, it will noticeably increase the dose to the patient due to its characteristic gamma-rays (1,065.1 keV 10.8%, 1,154.1 keV 10.4%, 1,222.4 keV 31%, 1,421.7 keV 12.2%, etc.). However, dosimetry calculations showed that the use of ^155^Gd enriched targets, containing ^156^Gd as impurity ≤2%, keeps the dose increase below the 10% threshold and ensures high-quality images (80).

A deuteron-induced reaction on ^155^Gd, enriched to 92.8%, for ^155^Tb production was recently performed at GIP ARRONAX cyclotron facility (20). The target material also contained 5.7% ^156^Gd. The cross-section peak for the reaction *Gd(d,x)^155^Tb is almost 800 mb at the deuteron energy ∼14.2 MeV. However, the cross-section of ^156^Tb production at the same point was 83 mb, and ^154g/m1/m2^Tb radionuclides were detected. As a result, maximum purity of ^155^Tb even after 14 days of cooling was not more than 89%. The production yield at the End of Bombardment (EoB) was determined to be 10.2 MBq/µAh at deuteron beam energy of 15.1 MeV, which may be enough for preclinical studies. It may have great potential if the ^156^Gd content of the enriched ^155^Gd target does not exceed 2% (80).

**Figure 3:**
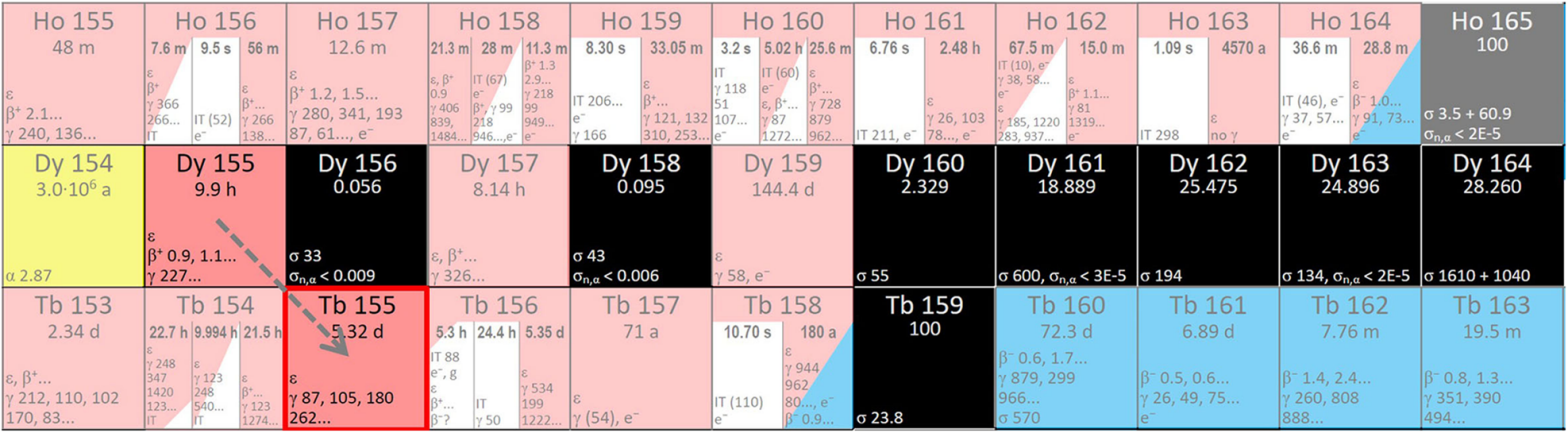
Region of the Karlsruhe nuclide chart displaying possible Dy to Tb nuclear reactions (www.nucleonica.com).

Alternatively, ^155^Tb can be produced using Tb_4_O_7_ targets via an indirect production route [^nat^Tb(p,5n)^155^Dy → ^155^Tb] (45, 81, 82). In this way, as a decay product of ^155^Dy, radionuclidically pure ^155^Tb can be produced (42, 43). ^155^Dy has a compatible half-life (9.9 h) and decays into ^155^Tb (Figure 3). Furthermore, neighbouring dysprosium nuclides do not decrease the purity of the product in the case of ^155^Tb separation from the Dy fraction.

Several other indirect methods were researched, e.g., photon irradiation of ^156^Dy targets would lead to ^155^Tb production (58). However, the abundance of ^156^Dy in the natural material is only 0.056%, which means that the production of a monoisotopic ^156^Dy target is likely to be unaffordable. Also, the yield of the photonuclear reaction is 3.0 µCi/(µAh·g) e.g., 111 kBq/(µAh·g), so it is not enough for medical purposes.

Finally, promising results were presented by the Kurchatov Institute with irradiation of gadolinium targets with α-particles (55, 57). ^nat^Gd and enriched ^155^Gd targets were irradiated with 60 MeV and 55 MeV α-particles, respectively, to reach the ^nat^Gd(α,*x*n)^155^Dy → ^155^Tb and ^155^Gd(α,4n)^155^Dy → ^155^Tb nuclear reaction. In the case of the natural target, the thick target yield (TTY) of ^155^Tb was 3 MBq/μAh, and the terbium fraction had 94.6% RNP after two separation processes. The only impurity was ^153^Tb (5.4%), due to the parallel nuclear reactions on ^152,154^Gd, leading to the formation of ^153^Dy (T_1/2_ 6.4 h, EC) and, as a result, ^153^Tb in the product (55, 56). Irradiation of enriched material allowed the production of ^155^Tb with RNP of >99.5% and the only radionuclidic impurity was ^156^Tb. However, the result can be improved to >99.99% through radiochemical separation, as the α,4n reaction on ^156^Gd yields stable ^156^Dy and no protons for the direct nuclear reaction are available to produce ^156^Tb. Any terbium impurities can be separated radiochemically in the initial separation step from ^155^Dy (57). The TTY of the reaction ^155^Gd(α,4n)^155^Dy → ^155^Tb (11.7 MBq/μAh) is comparable to the ^156^Gd(p,2n)^155^Tb reaction (12.7 MBq/µAh). However, an α-particle accelerator capable of reaching 40 MeV or higher is required.

Nuclear reactions induced by α-particles and ^3^He-particles may also be used for ^149^Tb and ^152^Tb production (21, 22). ^149,152^Tb were produced using both types of irradiations, but the reaction with ^3^He-particles resulted in a low TTY of ^152^Tb, and the cross section of the reaction ^151^Eu(α,6n)^149^Tb was not more than 10 mb in the investigated energy range. However, the excitation function of ^151^Eu(^3^He,5n)^149^Tb reaction reaches 70 mb at 47 MeV (21). Thus, for 8 h irradiation with a current of 20 μA, 3.4 GBq (EoB) of ^149^Tb would be produced. Radionuclidic impurities such as ^150,151^Tb would also be formed simultaneously. The optimal energy range was calculated according to an optimal ratio TTY/RNP to increase RNP, and irradiation of a 670 µm ^151^Eu_2_O_3_ target was proposed as a reasonable compromise. In this case, the output energy of ^3^He-particles would be ∼40 MeV, TTY of ^149^Tb 150 MBq/μA, and RNP ∼43%. The main radionuclidic impurity (∼100%) would be ^150^Tb (T_1/2_ = 3.48 h, EC β^+^ 100%), which may have no critical negative biological effects for the dose load due to its half-life, decay type, and γ-ray emission. The second radionuclidic impurity (28%), ^151^Tb (T_1/2_ = 17.609 h, EC β^+^ 100%), is undesirable due to its hard γ-ray emission. Nevertheless, the method can be useful due to the high ^149^Tb production yield, suitable at least for preclinical studies. Should one wish to pursue clinical application using this route, mass-separation techniques may need to be used.

In addition, ^151^Eu irradiation with α-particles can be an interesting route to produce ^152^Tb. Indeed, the cross-section peak of the ^151^Eu(α,3n)^152^Tb reaction reaches 800 mb, would make it possible to produce up to 8 GBq with an 8 h irradiation at a current of 20 μA (22). ^151,153^Tb radionuclides are also formed during the irradiation, but it is impossible to reduce their activity when irradiating in the maximum cross-section energy range. Thus, the optimal energy range was proposed as 42 → 34 MeV, with the TTY of ^152^Tb equal to 222 MBq/μA for 8 h irradiation. In this case, the total content of impurities would be less than 20%: ∼9% of ^151^Tb and ∼9% of ^153^Tb. However, ^151^Tb is an undesirable impurity and can be eliminated by decreasing the energy of incident α-particles. Due to the *Q*-value for the reaction ^151^Eu(α,4n)^151^Tb, equal to −32.89 MeV, it is possible to avoid ^151^Tb production with α-particles with energy <35 MeV. As a result, the only radionuclidic impurity would be ^153^Tb (T_1/2_ = 2.34 d, EC β^+^ 100%) with only one high-intensity γ-ray line at 212.00 keV (28%). However, its influence on biological studies requires further research. In addition, using the abovementioned conditions, the TTY of ^152^Tb would decrease from 65.7 MBq/μAh to ∼30 MBq/μAh.

### 2.3 Heavy-ion nuclear reactions

Heavy-ion (^7^Li, ^10,11^B, ^12^C, ^14,15^N, ^16,18^O, ^19^F, ^20,22^Ne) reactions provide means to access radionuclides that are located far from the stability line. Thus, this method can be suitable for ^149^Tb and, to a lesser extent, for ^152^Tb production.

Over the years, five institutes explored ^149,152^Tb production from target irradiation with heavy ions (67, 68, 73, 75, 83–88). However, the interest in such research faded in the 2000s. The main reason was due to the low yield and purity of the product, as well as the complex radiochemical separation process necessary. The last publication covering this type of nuclear reaction was produced more than a decade ago (88), while other attempts to isolate Tb from targets irradiated with heavy ions (published more than 20 years ago) were not fully successful (68, 73).

The initial goal of the earlier studies was only to investigate physical aspects of the formation of the ground and metastable states, since ^149m^Tb does not decay into ^149^Tb. Thus, the ground state ^149g^Tb can only be formed as a result of a direct nuclear reaction, and individual cross section thereof can be calculated and compared with theoretical data. Numerous reaction cross sections were presented for ^10,11^B, ^12^C, ^14,15^N, ^16,18^O, ^19^F, ^20,22^Ne beams in the energy range from 150 MeV to 35 MeV on target materials ranging from Ba to Nd (75, 83, 84). According to the results, all the reactions M(HI,*x*n)^149,152^Tb studied, where M is the target material, HI are heavy ions, yield low activities of ^149g^Tb. The largest value of cross section for the formation of ^149g^Tb turned out to be in the ^142^Nd(^11^B,4n)^149g^Tb nuclear reaction, which resulted in ∼58 mb at an average energy of 55.2 MeV ^11^B ions (75), much lower than that theoretically calculated. In contrast, the excitation function of ^149m^Tb (T_1/2_ = 4.16 min) seems to be much larger than that of the ground state for the same nuclear reaction on Nd. It was also noted that the ratio of the probability of formation of ^149m^Tb to ^149g^Tb increases very quickly with increasing energy. Therefore, it can be concluded that ^149m^Tb has a higher spin than ^149g^Tb, and an isomeric transition is unlikely to happen (75)*.* Although direct reactions of ^149g^Tb production with heavy ions have a low yield, an indirect route via ^149^Dy is of interest. ^149^Dy (T_1/2_ = 4.20 min) decays into ^149^Tb, and cross sections for the formation of ^149^Dy with the participation of heavy ions are much higher than for ^149^Tb (85). The reaction cross section for the formation of ^149^Tb from ^142^Nd reaches 446 mb at an incident particle energy of 97 MeV (67). It would allow the production of 15-30 GBq ^149^Tb from enriched targets of ^142^Nd (∼60 mg/cm^2^ thick) after 8–10 h bombardment with ^12^C ions at an energy of 120 MeV and an intensity of 50–100 μA (73, 86, 87).

On the other hand, regarding ^152^Tb, an irradiation of a thick ^nat^Nd target with ^12^C nuclei at an energy of 85 MeV for 15.3 h would allow production of 100 MBq ^152^Dy at EoB (68). The RNP of the daughter radionuclide ^152^Tb should be high enough for biological studies, however, that was not measured. ^144^Sm targets have also been irradiated with ^7^Li ions to produce ^152^Tb, but the cross-section peak was only 45 mb (88).

### 2.4 Spallation reaction

The spallation reaction by itself cannot produce a pure mononuclidic product. However, when used in combination with ISOL techniques, ^149^Tb and its parent radionuclide ^149^Dy (T_1/2_ 4.20 min) can be produced by spallation of suitable target with high cross-section (70). The proton-induced spallation reaction occurs with high-energy protons on the desired target material. The spallation products produced (a vast quantity of nuclides) are released and extracted at high temperature (over 2,000°C). The resultant ion beam is accelerated and mass-separated online in a magnetic sector field and the desired mass number collected by implantation into a foil (the ISOL technique). The desired radionuclide is subsequently chemically separated from its isobars and impurities. In addition, diagnostic ^152,155^Tb can be produced with this method as well. For this purpose, cyclotrons accelerating protons to hundreds of MeV energies are required.

The first attempts to produce ^149^Tb using a spallation reaction were carried out in the 1960s (72, 89–91) and continued for the next five decades (71, 92). Tantalum, gold, and bismuth targets were irradiated with high-energy protons (up to 30 GeV). The production of ^149^Tb was used as a monitor reaction to estimate the proton beam current due to easily identified α-particles of ^149^Tb. A rapid increase in the cross section of this reaction on a gold target in the energy range of 0.2–0.5 GeV was shown (90). It peaks at 1.3–2 GeV (70) and then up to 30 GeV, the cross section of this reaction decreases slightly (72, 89). The cross-section peak (∼19 mb) of the spallation reaction on a tantalum target is reached at a proton energy of 1–1.7 GeV, and on a bismuth target—2.0–3.2 GeV (∼10.5 mb) (72). Thus, by the time nuclear medicine became interested in terbium radionuclides (5, 31), the production of ^149,152,155^Tb had already been demonstrated at ISOLDE/CERN, Switzerland, using spallation reactions on tantalum target (12, 15, 93). The spallation method is very effective, but so far, there are few large facilities built for this purpose. However, recently, successful ^155^Tb production at the ISAC facility (TRIUMF, Canada) was published (94), while new facilities in Belgium (ISOL@MYRRHA), Switzerland (IMPACT-TATTOOS), and Japan (J-PARC ISOL) have been announced for launch over the next decade (10).

## 3 Separation of terbium sisters from irradiated target

Due to the numerous options for producing terbium radioisotopes, methods to isolate them from various lanthanides, such as lanthanum, cerium, praseodymium, and neodymium, as well as europium, gadolinium, and dysprosium have been reported.

In the past, liquid-liquid extraction was proposed to separate terbium isotopes from a lanthanum or cerium target irradiated with ^16^O nuclei, as well as from a neodymium target irradiated with ^12^C nuclei (66, 95, 96). A solution, containing the target dissolved in HNO_3_ + H_2_SO_4_, was evaporated and redissolved in HCl. A hydrochloric acid solution was equilibrated with a solution of di(2-ethylhexyl)phosphoric acid (HDEHP) in cyclohexane. Separation factors with different concentrations of HDEHP and HCl were studied. The optimal concentrations for the three systems are shown in Table 5.

**Table 5:** Conditions for liquid-liquid extraction of terbium from La, Ce, and Nd targets (66, 95, 96).

Target material	HDEHP concentration	HCl concentration
La	1%	0.1 M
Ce	10%	0.001 M
Nd	10%	0.1 M

About 70% of the terbium fraction transitioned into the organic phase in one cycle, completely retaining the macro amount of target material in the aqueous phase. A high separation factor (∼800) was achieved for the lanthanum/terbium system. However, solvent extraction is less effective for the separation of neighbouring lanthanides, as it typically requires multiple stages to achieve separation. As a result, cerium/terbium and neodymium/terbium separation were complicated by the presence of dysprosium isotopes (lanthanide adjacent to terbium), thus, requiring additional separation. Multiple extraction and back-extraction of dysprosium into the aqueous phase in a 10% HDEHP/1.5 M HCl system allowed the terbium fraction to be isolated in the organic phase (with 10% loss of terbium re-extracted into the aqueous phase along with dysprosium). A complete re-extraction of terbium fraction from the organic phase was possible with an aqueous solution of 1 M HCl. Reverse recovery of lanthanum radioisotopes has been studied using DTPA and EDTA solutions so that they could be directly applied *in vivo* without further processing (95). The need for repeated back and forth extractions to separate neighbouring lanthanides by liquid-liquid extraction make this approach inefficient. Subsequently, extraction resins were developed and have been used for this process to improve on separation efficiency.

Nowadays, the most useful method for lanthanide separation is by cation exchange chromatography, as strong cation exchange resins are specifically helpful for separating neighboring lanthanide pairs. Cation exchange resins operate by exchanging positively charged lanthanide ions with cations on the resin, where smaller, heavier lanthanides with higher charge densities are exchanged and eluted earlier due to stronger electrostatic interactions. For example, a separation of terbium fraction from irradiated neodymium target was provided using a chromatographic column filled with KU-2 or DOWEX 50 cation exchange resin (a copolymer of sulfonated divinylbenzene and styrene) (73). Before the elution of terbium, the column was saturated with 1.2 M NH_4_Cl. Terbium was eluted from the column with α-hydroxyisobutyric acid (α-HIBA), with yield >90%.

A similar method was proposed for a separation of ^149^Tb from the mix of isobars collected after mass separation of a tantalum target irradiated with protons (87). The solution containing 149 isobars included ^149^Eu, ^149^Gd, ^149^Tb—as well as pseudo-isobars ^133^La and ^133^Ce collected as ^133^LaO^+^ and ^133^CeO^+^. All lanthanides in chloride form were separated on a column, filled with AMINEX A5 cation exchange resin (KU-2 analogue) and afterwards eluted with α-HIBA at pH 5.0 using a concentration gradient, resulting in a terbium elution yield exceeding 90%.

Unfortunately, the AMINEX A5 and A6 resins are not produced anymore, but Bio-Rad has replaced them with alternatives in limited supply. Other strong cation exchange resins may be used to separate lanthanides (14, 24). An extraction chromatographic separation method on LN resin was proposed for the production of ^161^Tb as an alternative to cation exchange resins (37, 38, 97, 98). Commercially available LN Series Extractants (Triskem, Eichrom) based on HDEHP were deemed suitable for lanthanide separation. Unlike cation exchange resins, elements with smaller atomic numbers are washed off from LN resin earlier than those with greater atomic numbers. This is due to the extraction mechanism of the LN resin, where heavier lanthanides, possessing smaller ionic radii and higher charge densities, form more stable complexes with the extractant ligands on the resin, resulting in delayed elution. Therefore, in the gadolinium/terbium system, the gadolinium fraction is washed off earlier than the terbium fraction. This can affect the purity of terbium fraction, as the long “tail” of the gadolinium elution profile may overlap with that of terbium, especially when considering upscaling the method to handle larger gadolinium targets. Nevertheless, the terbium/gadolinium separation factor was demonstrated to be the highest for lanthanide pairs and the method can be implemented for terbium production. LN resin was also used in a two-step separation of terbium from gadolinium targets irradiated with α-particles (55, 57), where a dysprosium/gadolinium separation was conducted, followed by a dysprosium/terbium separation (after decay of ^155^Dy into ^155^Tb). In particular, the ^155^Dy produced by the irradiation was separated from the gadolinium target, followed by the isolation of ^155^Tb obtained from ^155^Dy decay 40 h later. When irradiating enriched (90.4%) ^155^Gd, a less efficient dysprosium/terbium separation was observed, which led to <0.3% ^156^Tb in the ^155^Tb fraction. In another work, the separation yield of terbium after isolation from a 20 mg dysprosium target with LN resin yielded only 39% separation efficiency (29). These discrepancies showcase that optimal parameters for terbium/dysprosium separation still need to be devised. In addition, LN3 resin was proposed for post-purification and concentration of terbium fraction in 0.05 M HCl (19).

Later, a study on ^161^Tb radiochemical separation with the more recently developed TK211 and TK212 resins was published (33). These resins are similar to LN resins but use mixed organophosphoric, organophosphonic and organophosphinic acid extractants that may work in synergy to improve selectivity (99). Using these types of resins, it was possible to separate ^161^Tb and Dy from the gadolinium fraction on TK212 resin and then isolate the Tb fraction on TK211 resin. Moreover, a semi-automated module for the proposed separation system was built. Quality controls on the terbium fractions showed nanogram levels of gadolinium, however, that did not affect terbium labelling capabilities. This could be used for the reprocessing of aged/partly decayed ^161^Tb by separating it from its stable daughter (^161^Dy), which interferes with apparent molar activity and, as a result, chemical purity.

In conclusion, there are currently three methods for the selective isolation of terbium from irradiated lanthanide targets and their decay products (Table 6).

**Table 6:** Proposed methods for Tb separation from irradiated lanthanide target.

Resin	Base	Eluent	Advantages	Disadvantages
Strong cation exchange resins (Sykam/Aminex A5/KU-2)	Sulpho-cationite	α-HIBA	Elution of Tb before Gd target material, high loading capacity, high radiation stability, high separation factors for Tb/Gd and Tb/Dy, chemically anchored functional groups	Commercial unavailability, high pH dependence, use of α-HIBA that is undesirable for nuclear medicine, high sensitivity to eluent molarity
LN/LN2/LN3	HDEHP/HEH[EHP]/H[DTMPP]	HCl	Commercial availability, possibility to get fraction in desirable for nuclear medicine solution (0.05 M HCl), high separation factor for Tb/Gd	Low radiation stability, high commercial price, elution of Gd target material before Tb, lower capacities compared to cation exchangers, possible washing off of the impregnated organic layer
TK211/TK212	Different mixtures of organo-phosphoric, organo-phosphonic, and organo-phosphinic acids	HNO_3_	Commercial availability, possibility to get fraction in desirable for nuclear medicine solution (0.05 M HCl), high separation factor for Tb/Gd and Tb/Dy,	High commercial price, necessity to convert HNO_3_ solution into HCl, elution of Gd target material before Tb, lower capacities compared to cation exchangers, possible washing off of the impregnated organic layer, additional experiments are needed (radiation stability, mass, dimension, flow rate, etc.)

The authors believe that the main problems for terbium sisters’ production nowadays include the poor availability of separation components (especially chromatographic resins with suitable specifications), as well as the limited supply of enriched target material. HDEHP-based resins are expensive and are being produced by few manufacturers. Moreover, extraction resins also have a limited loading capacity. The physical impregnation of the organic layer onto the solid support limits the lifetime of using these resins for neighbouring lanthanide separations. Therefore, given the potential of the terbium sisters in the field, it is of paramount importance to increase the availability of resins and separation method possibilities for terbium radioisotope production.

## 4 Opportunities and potential challenges of using terbium sisters

**Figure 4:**
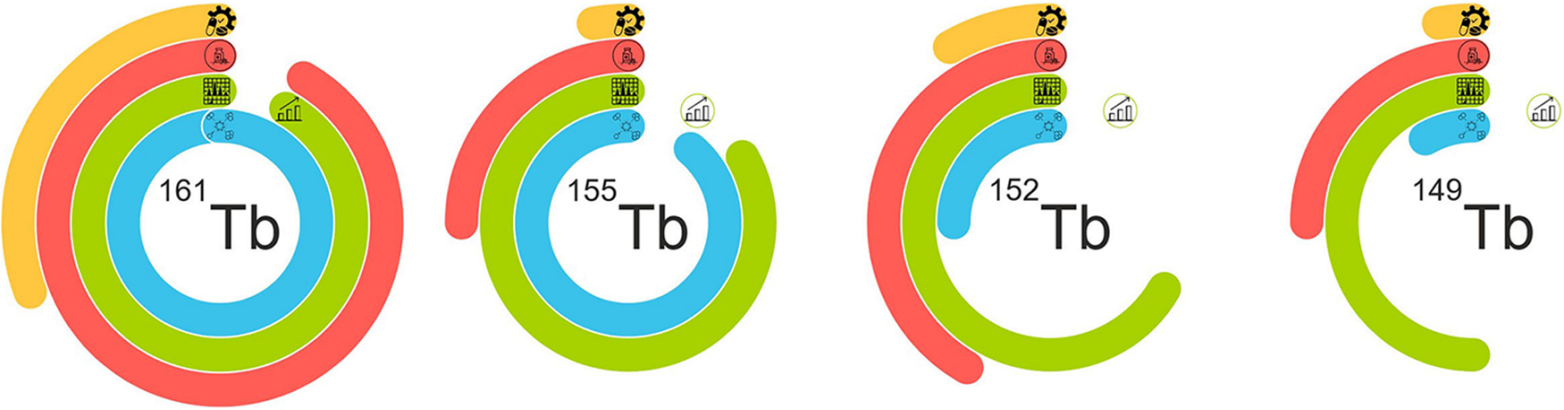
Representation of the current situation of terbium sisters’ production. Four rings symbolize the four major phases of radiopharmaceuticals implementation: the blue line shows cyclotron/reactor production situation, the green line represent separation process readiness and includes problems with the scale-up process at its last bend, the red line shows preclinical studies, and the yellow line–GMP development and clinical implementation.

The current production situation for terbium sisters is summarized in Figure 4.

### 4.1 Terbium-161

A lot of work has been done towards ^161^Tb production since its initial publication (30) over a decade ago (Figure 4). Nevertheless, there are still considerable tasks remaining to be addressed. The most optimal way to produce ^161^Tb is via indirect method ^160^Gd(n,γ)^161^Gd→^161^Tb. The main challenge of this method is the chemical purity of the target material, as it needs to contain less than 5 ppm of ^159^Tb (30) in order to decrease ^160^Tb production via the ^159^Tb(n,γ)^160^Tb nuclear reaction and ensure that the radionuclidic and radiochemical purity of the resulting ^161^Tb exceeds 99% (24, 37). Another challenge to be faced in the long term is the availability of highly enriched target material, since the chains of nuclear reactions ^158^Gd(n,γ)^159^Gd → ^159^Tb(n,γ)^160^Tb and ^157^Gd(n,γ)^158^Gd(n,γ)^159^Gd → ^159^Tb(n,γ)^160^Tb are also contributing to the final content ^160^Tb in the final product. Three successful separation methods were developed: using macroporous cation exchange resin [up to 140 mg of ^160^Gd_2_O_3_ (24)], using LN resin [up to 5 mg of ^160^Gd_2_O_3_ (38)], and using TK212/TK211 resins [up to 10 mg of Gd_2_O_3_ (33)]. Currently, the primary focus for ^161^Tb revolves around its medical applications. Clinical trials should be conducted to benchmark it against ^177^Lu. In the meantime, ^161^Tb production must be upscaled to provide enough activity for clinical studies. For that, a few changes in the current separation process should be implemented (for example, a lengthy evaporation process should be avoided to optimize production time). To date, the first studies in humans with radiopharmaceuticals based on ^161^Tb were started at the Universitätsspital Basel, Switzerland (79, 100), Saarland University, Medical Centre, Germany (101), King Hussein Cancer Centre, Jordan (102), and Peter MacCallum Cancer Centre, Australia (78).

### 4.2 Terbium-155

Terbium-155 has several promising methods for its production and separation, but they have yet to be improved. However, it is assumed that the medical implementation of other terbium sisters might occur much faster than ^161^Tb due to the identical chemical properties of all terbium radionuclides (31, 34). Nowadays, two methods are mainly being used for ^155^Tb production. The first is the spallation reaction on a tantalum target followed by ISOL separation. This method was realized by CERN and TRIUMF at the scale of hundreds of MBq (31, 34). Furthermore, radiochemical separation of ^155^Tb from isobars is required. Despite the consumption of a lot of energy and materials this process could provide the activity only for preclinical studies and cannot be implemented in routine medical cycles. Another method is proton irradiation of gadolinium targets enriched in ^155^Gd or ^156^Gd (19, 39). The ^155^Gd(p,n) reaction gives a purer product (RNP up to 94%), but the yield of the reaction is twice lower than the ^156^Gd(p,2n) reaction (RNP up to 92%). The recently studied ^155^Gd(d,2n)^155^Tb nuclear reaction may become the third method for ^155^Tb production (RNP up to 89%) (20). In all these scenarios, however, the produced impurity is ^156^Tb—an undesirable radionuclide for nuclear medicine which has a comparable half-life to ^155^Tb (5.35 days) and could noticeably increase the dose to patient due to abundant hard γ-lines. To prevent the presence of ^156^Tb in the ^155^Tb solution, an ultra-pure ^155^Gd or ^156^Gd target may be used, which drastically affects the material cost.

Otherwise, two indirect methods via ^155^Dy production were proposed (45, 55). The idea of indirect production of ^155^Tb is being actively studied at the PSI and the Kurchatov Institute (42, 55, 57). These two institutes have different large facilities, so the production methods will differ. However, both are based on the production of ^155^Dy, followed by its quick separation from the target material and final isolation of ^155^Tb after ^155^Dy decay. The radiochemical separation technique proposed by the Kurchatov Institute (separation of ^155^Dy from ^155^Gd targets irradiated with α-particles, and then ^155^Tb from the ^155^Dy fraction) should be simpler than separation of ^155^Dy from ^nat^Tb, but in this case, an α-accelerator is required. Proton accelerators have better distribution, the yield of ^155^Tb should be ten times higher in the case of proton irradiation, and the use of natural material more convenient. Unfortunately, the separation of terbium and dysprosium appears to present greater challenges. As a result, the optimal production and separation methods for ^155^Tb are still under development.

In conclusion, increased investment in large-scale facilities with the necessary capabilities to conduct these reactions is crucial for advancing the study of promising indirect production routes. Such infrastructure would enable higher yields and more efficient production processes. Additionally, the radiochemical separation between terbium and dysprosium should be deeply investigated, as this separation is more challenging compared to the well-documented terbium/gadolinium separation. Addressing this challenge is essential for improving the production purity of ^155^Tb. Furthermore, the successful clinical application of ¹⁵⁵Tb will rely not only on optimized production and separation techniques but also on a comprehensive understanding of radiation effects and precise dosimetry for both patients and healthcare personnel. These factors must be rigorously evaluated to ensure the safe and effective use of ¹⁵⁵Tb in medical applications.

### 4.3 Terbium-152

Medical implementation of ^152^Tb was studied better than ^155^Tb, due to the urgent need for additional β^+^-emitters (13). ^152^Tb can be produced via the spallation reaction and the ISOL collection method (31), or the ^152^Gd(p,n) reaction (63). Unfortunately, the available enrichment of ^152^Gd (30% maximum) is too low to ensure a radionuclidically pure product. Therefore, ^152^Tb's success will depend on ISOL facilities being constructed in the future as nowadays, this production method is very limited. In this context it is expected that ^152^Tb production will see significant advances at the PSI with the TATTOOS development (Targeted Alpha Tumour Therapy and Other Oncological Solutions, https://www.psi.ch/en/impact/tattoos) as part of the upgrade of the High-Intensity Proton Accelerator complex HIPA which plays a pivotal role in Swiss large-scale infrastructure. HIPA's Ring Cyclotron is the most powerful proton cyclotron worldwide and among the most energy-efficient accelerators. It provides a world-leading 1.4 MW high-intensity proton beam of 590 MeV energy and up to 2.4 mA of beam current to target stations. According to the calculated data, 12 h irradiation of a tantalum target on TATTOOS (100 *μ*A, 590 MeV proton beam) gives 625 GBq of ^152^Tb (103). Due to the 50-fold higher proton beam intensity foreseen at TATTOOS (100 μA) compared to ISOLDE/CERN (2 μA), this corresponds to an at least 50-fold increase compared to ISOLDE's production capacity, under consideration of all extraction and adsorption losses as well as losses during ionization and ion transport. A similar setup is planned at ISOL@MYRRA (Belgium, https://myrrha.be/myrrha-applications/nuclear-science/isolmyrrha), which will allow for extended preclinical and clinical studies. The ISOL method will allow to use of the same radiochemical separation system, that already was proposed for ^152^Tb isolation from isobars (12, 13). As for other terbium radionuclides collected with isobars, cation exchange chromatography is a rapid and effective way to isolate them.

### 4.4 Terbium-149

Finally, great interest in ^149^Tb has been shown by nuclear medicine physicians, due to its alpha emission (104). Its production and separation are provided with the same methods, as for ^152^Tb. Unfortunately, the production of ^149^Tb is the most challenging, as there are no known nuclear reactions capable of yielding a radionuclidically pure product. As a result, offline mass-separation or ISOL techniques are inevitable, but the production yield should be improved, which, as mentioned above, should be feasible in facilities with higher currents such as TATTOOS or ISOL@MYRRHA. The calculated production yield of ^149^Tb as obtained from TATTOOS using a tantalum (12 h irradiation) target gives 2,390 GBq of ^149^Tb. The radiochemical separation method from isobars was also previously reported and based on cation exchange resins (31, 70). Although the therapeutic activity has yet to be established for ^149^Tb-based preparations, one can estimate it from other alpha emitters. For ^213^Bi this value is 10–50 MBq/kg; for ^225^Ac—20–150 kBq/kg, for ^223^Ra–55 kBq/kg, and for ^212^Pb, a parent of ^212^Bi,—200–500 kBq/kg (105). It can be assumed that the therapeutic activity of ^149^Tb can be 10–55 MBq/kg, according to its decay characteristics (α 16.7%, T_1/2_ = 4.118 h). Therefore, the activity produced with the abovementioned facilities would be more than enough for clinical application and even adequate distribution.

## 5 Conclusions & outlook

Discussions regarding the theragnostic potential of terbium sisters have persisted for over a decade. Nonetheless, significant progress has been achieved only with one terbium radionuclide, ^161^Tb. This is due mainly to difficulties associated with producing other terbium sisters with high RNP. ^155^Tb appears to be the most prospective diagnostic pair for ^161^Tb for the next decade due to more realistic production methods. Production of ^152^Tb and ^149^Tb requires the implementation of large facilities such as TATTOOS or ISOL@MYRRHA to ensure sufficient activity and RNP for medical purposes. Thus, large investments in such facilities are required, which are highly dependent on governmental funds and subsidies. Improved commercial availability of separation resins would expedite the development process and facilitate wider access to terbium worldwide. Therefore, in conclusion, significant investments should be directed towards large-scale facilities capable of producing such radionuclides in quantities suitable for clinical use and distribution. In this regard, collaborations of research centers, which has been initiated by The European medical radionuclides program (https://www.prismap.eu/), could be another valuable option to increase the availability of the terbium sisters for preclinical and clinical studies.
